# Fluorescence imaging of chromosomal DNA using click chemistry

**DOI:** 10.1038/srep33217

**Published:** 2016-09-13

**Authors:** Takumi Ishizuka, Hong Shan Liu, Kenichiro Ito, Yan Xu

**Affiliations:** 1 Division of Chemistry, Department of Medical Sciences, Faculty of Medicine, University of Miyazaki, Japan; 2 Research Center for Advanced Science and Technology, The University of Tokyo, Japan

## Abstract

Chromosome visualization is essential for chromosome analysis and genetic diagnostics. Here, we developed a click chemistry approach for multicolor imaging of chromosomal DNA instead of the traditional dye method. We first demonstrated that the commercially available reagents allow for the multicolor staining of chromosomes. We then prepared two pro-fluorophore moieties that served as light-up reporters to stain chromosomal DNA based on click reaction and visualized the clear chromosomes in multicolor. We applied this strategy in fluorescence *in situ* hybridization (FISH) and identified, with high sensitivity and specificity, telomere DNA at the end of the chromosome. We further extended this approach to observe several basic stages of cell division. We found that the click reaction enables direct visualization of the chromosome behavior in cell division. These results suggest that the technique can be broadly used for imaging chromosomes and may serve as a new approach for chromosome analysis and genetic diagnostics.

The study of human chromosomes provides valuable insight into processes of clinical diagnosis for many genetic disorders and analysis of chromosome architecture^1^,^2^,^3^. For example, more than 400,000 chromosome karyotype analyses for actual human genetic diagnoses are performed each year in the U.S. and Canada. The existing approaches of chromosome imaging tend to work effectively, but depend upon the binding of the fluorescent dyes with the chromosomal DNA. This condition raises a series of questions regarding the affinity of dyes to chromosomal DNA, the number of differently colored fluorescent dyes, and the photostability of the dyes^4^,^5^,^6^,^7^. Another important limitation is that, the shape variability of the chromosome, which is caused by the non-rigid nature of the chromosomal structure when placed on microscope slides, directly influences the interaction between the dyes and the DNA.

Furthermore, fluorescence *in situ* hybridization (FISH), a fundamental technology in many aspects of genetics, genomics, and cell biology^8^,^9^,^10^,^11^,^12^,^13^,^14^, is based on a mechanism whereby fluorescently labeled probes recognize and hybridize with the target chromosome DNA by nucleic acid base pairing. The technique enables researchers to identify rapidly the positions of genes and chromosomal aberrations in the clinic and research laboratory by determining the position of the fluorescent probe bound to the chromosomes. The success of FISH, wherein strained DNA is targeted by fluorescently labeled probes and then visualized via microscopy, depends on a specific state of the stained chromosomal DNA.

In addition, chromosomal staining is an essential experimental approach for the study of chromosome behavior in dividing cells^15^,^16^,^17^,^18^,^19^. Observing the basic stages of cell division by chromosome staining would provide the essential technology to better understand the significant biological process of cell division. Chromosomal staining in the series of stages of prophase of cell division requires a high-affinity binding between the dye and chromosomal DNA to provide clear visualizations. The traditional dye molecules used in the study of chromosomal behavior do not form covalent bonds with chromosomal DNA; therefore, it is difficult to monitor chromosome dynamics.

To overcome these limitations and further enable the potential of chromosome imaging to be fully exploited in both research and diagnostic laboratories, we have developed a chemistry-based strategy for imaging chromosomal DNA in multicolor in place of the traditional dye pairing.

Bioorthogonal chemical reactions have been achieved using the Staudinger ligation^20^, Diels-Alder reaction^21^, copper(I)-catalyzed azide–alkyne cycloaddition^22^,^23^,^24^,^25^,^26^, and strain-promoted azide–alkyne cycloaddition reactions^27^,^28^,^29^, which has allowed selective labeling of cellular proteins^30^,^31^,^32^,^33^,^34^,^35^, nucleic acids^36^,^37^,^38^,^39^,^40^,^41^,^42^,^43^,^44^, lipids^45^,^46^, and glycans^47^,^48^,^49^. Recently, based on the azide–alkyne click reaction, 5-ethynyl-2′-deoxyuridine (EdU) was reported as a thymidine analog to detect DNA synthesis in cells^38^,^39^,^40^. After its metabolic incorporation into DNA, EdU can be detected with fluorescent azides by click reaction. This method was used to label DNA in cell level by microscopic analysis^39^. An arabinofuranosylethynyluracil derivative (F-ara-EdU) also exhibited selective DNA labeling in cell level^38^. Using a click reaction, we successfully found an unusual nucleic-acid structure formed by DNA and RNA^50^.

Click reaction is a chemical reaction that can proceed in biological systems without interaction with the inside biomolecules or interference on the whole system^24^,^51^. The click reaction to form a covalent bond between the chromosomal DNA and fluorescent molecules with highly selective and reactive with in the biological environment, allows that chromosomal DNA imaging is not affected by interaction affinity and structure etc. On the contrary, the traditional dyes work in an affinity-dependent manner with the chromosomal DNA, the intracellular environment may influence the interaction of chromosomal DNA and ligands. A chemical reaction by a covalent bond to connect the chromosomal DNA and fluorescent molecules is believed to overcome this difficulty and is less affected by affinity, environment, or chromosomal structure.

Traditional DNA imaging methods have also used fluorescent fusion proteins, staining nucleic acids, immunostaining with antibodies^52^. All of these approaches are limited in terms of the low throughput due to their relatively large size of fluorescent proteins, the low cell membrane permeability of antibodies, and large perturbations to native systems. Click reaction, an enzyme-free approach to DNA imaging, would not only eliminate the need for enzymatic reaction, but also readily utilized the azide and alkyne groups by taking advantage of their small size and inertness to most components in a biological environment.

Despite advances in DNA labeling, the strategy has an undesirable characteristic related to the additional wash step was required to remove unbound, free fluorescent dyes.

Based on the results of the past studies, we have now extended this methodology to image chromosome DNA at an individual chromosome level. We developed a light-up (turn on) reporter strategy to stain chromosomal DNA. We designed and synthesized two new azidocoumarins as the pro-fluorophore that can produce a strong fluorescence in a click reaction. The azido group of the profluorophores quenches the fluorescence, but the azide-alkyne click reaction can eliminate the quenching, resulting in a strong fluorescence. The advantage of having emission dependent on the click reaction is that it allows us to stain chromosomes that do not require extensive wash steps to remove the unreacted fluorescent dyes in the cell. This method provides a high-resolution image allowing the visualization of individual chromosomes.

Next, we applied this strategy to FISH to identify telomeres in the end of chromosome. The chemistry-based method allows for determination of telomeres with high sensitivity and specificity.

We further extend the utility of this approach to study the chromosome states during cell division. Instead of the traditional dye staining, the click reaction enables direct visualization of several important stages in cell division.

## Results and Discussion

### Chromosome imaging using fluorescent azide molecules at the individual chromosome level

To image chromosomal DNA, an intramolecular-tagging approach is required^38^,^39^,^40^, because the target DNA sequences are typically not accessible for labeling within cells. We first introduced EdU into chromosomal DNA as a rapid labeling tag for a click reaction. Cells were labeled with 10 μM EdU for 2 h and reacted with Alexa488-azide. We observed very intense nuclear staining by a fluorescent azide, consistent with previous reports. In contrast, cells not labeled with EdU exhibited no detectable staining under the same reaction conditions with Alexa488-azide (Supplementary Fig. 1). These results provide direct evidence that EdU can be incorporated into cellular chromosomal DNA and that the labeling reaction is highly specific.

**Figure 1:**
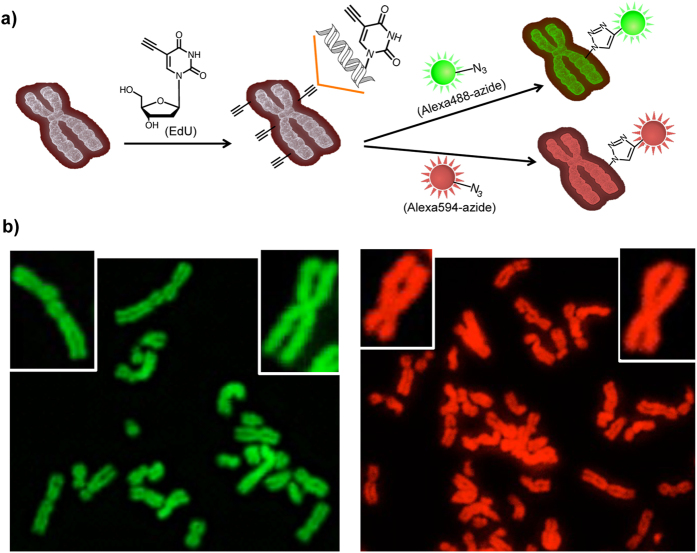
Chromosome staining by click raction. (**a**) Schematic of the click reaction for staining chromosomal DNA at the individual chromosome level. EdU was introduced into chromosomal DNA as a labeling tag for a click reaction. Alexa488-azide (green) or Alexa594-azide (red) reacted with EdU to stain the individual chromosome. (**b**) Chromosomes were stained with Alexa488-azide (green) or Alexa594-azide (red). The inset panel is at higher magnification. Observed by fluorescence microscopy.

To explore the possibility of a click reaction to stain individual chromosomes, cells were incubated with 10 μM EdU for 24 h and then stained with the fluorescent azides. We found that the click reaction produced uniformly stained chromosomes that could be visualized at the individual chromosome level with Alexa488-azide (green) and Alexa594-azide (red) (Fig. 1). We next stained chromosomes with multicolor imaging by using a click reaction. First, EdU-labeled chromosomes were reacted with Alexa488-azide. Subsequently, cells were stained with Alexa594-azide. The chromosomes are strongly stained for both the green and red colors (Supplementary Fig. 2).

We demonstrated that the fluorescent azide molecules react specifically and rapidly with the alkyne group of EdU to paint chromosomes. However, We found that for obtaining clear fluorescent image, the additional wash steps were required to remove unbound, free fluorescent dyes. Eliminating the repetitive wash steps to remove the unreacted fluorescent dyes resulted in a high level of background fluorescence and failed to observe nuclear staining (Supplementary Fig. 3).

### Preparation of two pro-fluorophore azidocoumarins 1 and 2 for chromosome imaging

**Figure 2:**
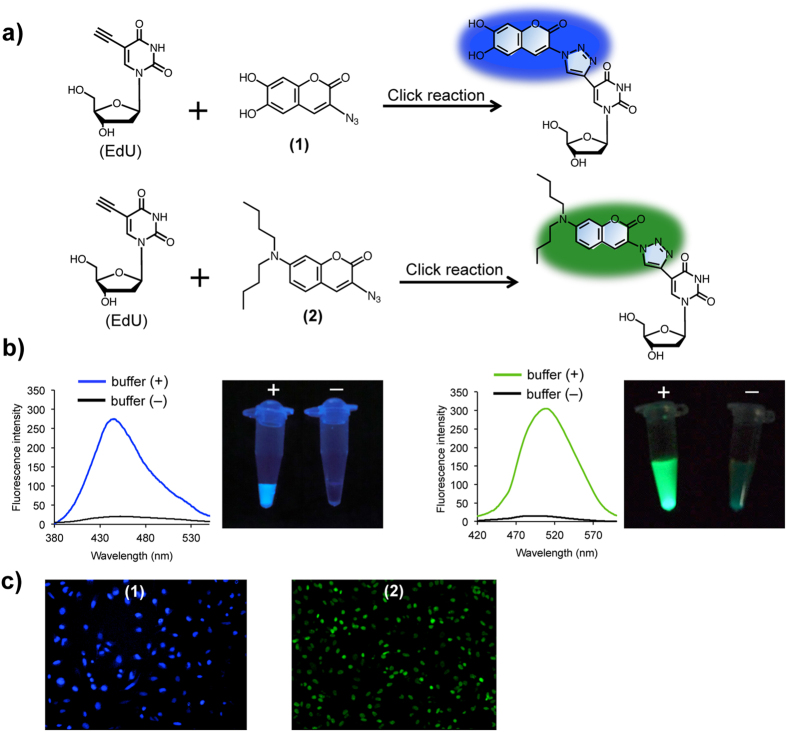
Two pro-fluorophores 1 and 2 for chromosome imaging. (**a**) Schematic of the click reaction between EdU with **1** or **2** served as a light-up reporter. The pro-fluorophores **1** and **2** containing azide moiety are fluorescent inactive. **1** and **2** reacted with EdU by the azide-alkyne click reaction can trigger the fluorescence signals. (**b**) (Left) Fluorescence spectra of **1** for click reaction with or without EdU and click reaction buffer. Fluorescence image with (+) and without (−) click reaction buffer after illumination with a UV lamp (365 nm). (Right) Fluorescence spectra of **2** for click reaction with or without EdU and click reaction buffer. Fluorescence image with (+) and without (−) click reaction buffer after illumination with a UV lamp (365 nm). (**c**) Cells were stained by 1 and 2.

To improve the labeling method and adapt it to ideal imaging of chromosomal DNA, We designed and synthesized two new pro-fluorophore azidocoumarins **1** and **2** from hydroxybenzaldehyde derivatives by a 3-nitro group conversion from a sodium azide reagent (Supplementary Scheme 1). The two new pro-fluorophores **1** and **2** have no fluorescence, because of the quenching effect of the electron-rich nitrogen in the azido group. Formation of a triazole ring at its 7-position by the azide-alkyne click reaction can eliminate quenching, resulting in a strong fluorescence (Fig. 2a)^53^,^54^,^55^,^56^,^57^,^58^. We performed fluorescence microscopy experiments to investigate the fluorescent properties of pro-fluorophores **1** and **2** (Fig. 2b and Supplementary Fig. 4). When only pro-fluorophores **1** and **2** were present in the solution, almost no fluorescence was observed; whereas, clear fluorescence observed with the naked eye after the addition of EdU via the click reaction (Fig. 2b). The fluorescence spectrum of **1** and **2** exhibits emission around 450 nm and 510 nm, respectively, after the click reaction (Fig. 2b). Importantly, the fluorescent product of **2** exhibits a red-shift in emission (from 450 to 510 nm), than that of **1**, which enables their specific emission wavelengths and eliminates the any background fluorescence in a specimen, a highly desirable feature for chromosome imaging.

We applied **1** and **2** to living cells and observed two clear colors, blue and green, in cells using a 360/40 nm excitation filter and a 470/40 nm emission filter for **1** (blue) and an excitation (480/40 nm) and an emission (527/30 nm) filter for **2** (green) (Fig. 2c). These results suggested that **1** and **2** are able to serve as light-up reporters to stain chromosomal DNA by click reaction. Next, we examined the time course of DNA light-up labeling in cells. We observed that the fluorescence intensities of **1** and **2** from the click reaction increased with time, reaching a plateau within 4 and 2 h for **1** and **2** (Supplementary Fig. 5), respectively, indicating a quick click reaction between **2** and cellular EdU-labeled DNA compared with **1**.

### Chromosome imaging using the pro-fluorophores 1 and 2

**Figure 3:**
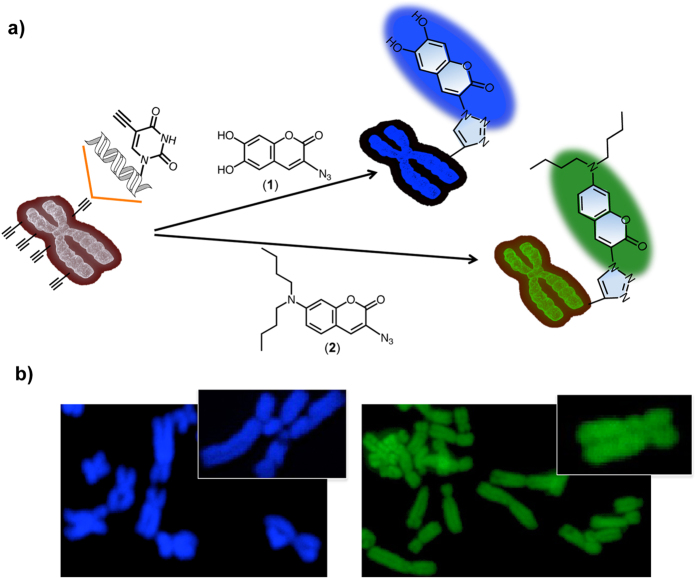
Chromosome imaging by using 1 and 2. (**a**) Schematic of a light-up (turn on) reporter strategy to stain chromosomal DNA. The pro-fluorophores **1** or **2** reacted with EdU-labeled chromosome to induce a strong fluorescence response for staining chromosome in blue (**1**) or green (**2**) color. (**b**) Chromosomes were stained with **1** (blue) or **2** (green). The inset panel is at higher magnification. Observed by fluorescence microscopy.

**Figure 4:**
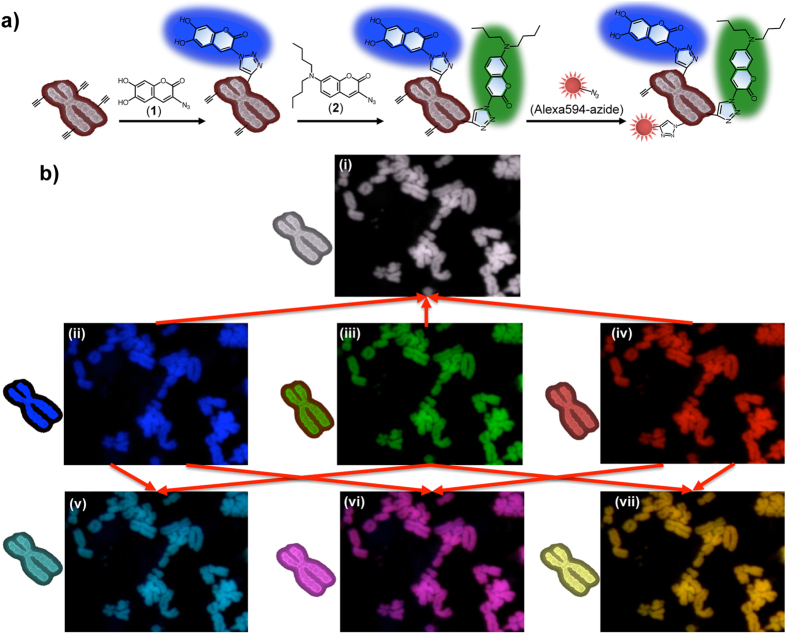
Staining chromosome with multicolor imaging by using click reaction. (**a**) Schematic of multicolor chromosome imaging by using a click reaction. First, **1** reacted with EdU-labeled chromosomes. Next, **2** reacted with the chromosomes. Finally, the chromosomes were stained by Alexa594-azide. (**b**) **1** staining in blue (ii), **2** staining in green (iii), Alexa594-azide staining in red (iv). Overlay of the **1** and **2** images shows in cyan (v). Overlay of the **1** and Alexa594-azide images shows in magenta (vi). Overlay of the **2** and Alexa594-azide images shows in yellow (vii). Overlay of the **1**, **2**, and Alexa594-azide images shows in white (i).

Having confirmed the efficient click reaction of **1** and **2** with EdU in cells, we were encouraged to apply this method to stain chromosomes with multicolor imaging. Cells were incubated with EdU and stained with 24 μM of **1** or **2**. We clearly visualized the chromosomes in green and blue colors (Fig. 3). To achieve multicolor painting chromosomes, we first stained cells with **1** for 4 h and then with **2** for 1 h. We obtained similar success in the chromosome visualization by assigning blue and green colors (Supplementary Fig. 6). To find a more useful application for chromosome staining, we combined **1**, **2**, and Alexa594-azide to image chromosomes (Fig. 4). The three staining patterns display a perfect fluorescent image (blue, green, and red). Multi-labeling offers a way to view images in an overlay mode, which displays a combined visible and fluorescence image. The two-color overlay allows us to visualize clearly the co-localization of two fluorescent probes in a specimen. The combination of **1** (blue) and **2** (green), **2** and Alexa594-azide (red), or **1** and Alexa594-azide is represented in the cyan, magenta, and yellow images (Fig. 4), respectively. Further, the three-color overlay produces (**1**, **2**, and Alexa594-azide) a white fluorescence image. Observing the chromosome in the desired color is possible by changing the fluorescent dye molecules that react with the DNA and achieving an overlay mode, suggesting that the click reaction strategy provides a useful tool for imaging chromosomes.

### Advantage of the click reaction method compared to traditional method

By using the turn-on fluorescent strategy via the click reaction, having eliminated the background fluorescence which remained a matter of concern from traditional dye staining, we carried out two comparison experiments to further demonstrate the advantage of the click reaction method compared to traditional dye painting. We first used propidium iodide (PI), which was the most widely used traditional dye, to strain chromosomal DNA. Although uniform fluorescence of chromosomal arms was produced, additional wash steps that are required to remove unbound, free fluorescent dyes, can result in loss of signal. We found that the repetitive wash steps to remove unbound dyes (10 times) induce unclear chromosomal DNA signals in traditional method, but the chromosomal DNA strained by click reaction method could be clearly observed even when the wash steps were added to 20 times (Supplementary Fig. 7a). Next, we compared the click reaction method with traditional method in multicolor chromosome imaging. The chromosomal DNA stained with PI (red) was subsequently bound with Hoechst (a blue fluorescent dye). We visualized the chromosomes in red color with PI, but the clear chromosomes were unable to be observed in blue color with Hoechst (Supplementary Fig. 7b). The two-color overlay does not also produce a clear chromosome DNA in a desired color (Supplementary Fig. 7b). The traditional dyes work in a binding-dependent manner with the chromosomal DNA, leading to results that the affinity of dyes to chromosomal DNA is very sensitive to DNA conformation and chromosome state. The dye PI that already binds to chromosome DNA at first binding step is believed to affect the secondary dye (Hoechst) binding with DNA. On the contrary, the click reaction connects the chromosomal DNA and fluorescent molecules by a covalent bond and allows chromosomal DNA imaging that is not affected by affinity, environment, or chromosomal structure. These results provide a new approach to overcome the technical limitations in traditional chromosome staining.

### Click reaction application in FISH assay

**Figure 5:**
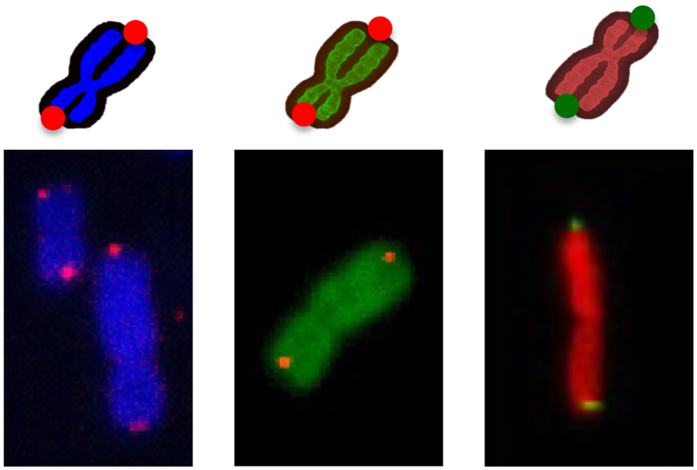
Chromosomes stained by click reaction for FISH assay. Chromosomes were stained with **1** (blue), **2** (green), and Alexa594-azide (red). Red and green signals at the ends of each chromosome are fluorescent-labeled FISH probes for repetitive TTAGGG telomere sequences.

Encouraged by these data, we next tested whether highlighting the chromosomes with this method would be effective with the FISH technique. FISH uses fluorescent probes to bind only parts of the chromosome by means of sequence complementarity. FISH is often used to find specific features in DNA for use in genetics, medicine, and species identification, by determining where fluorescent probes are bound to the paired chromosomes. We applied this method to detect human telomeres at the end of the chromosomes, a commonly-used target in FISH^59^,^60^,^61^. We directly detected the telomere from **1**, **2**, and Alexa594-azide labeled human chromosomes by FISH (Fig. 5). We observed the red and green fluorescent spots at the ends of each chromosome from **1**, **2**, and Alexa594-azide labeled human chromosomal DNA (blue, green, and red), when fluorescently labeled FISH probes for telomere DNA were fluorescent Cy3 (red) and FAM (green). These results demonstrated that chromosomes stained using click reaction provide a highly applicable substitute for FISH.

### Click reaction for application in the study of cell division

**Figure 6:**
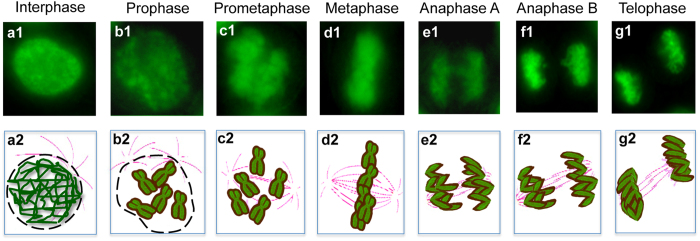
Chromosome behavior in the various stage phases of cell division. (panels a1–g1) Indirect fluorescence of chromosome DNA stained by click reaction and schematic representation (panels a2–g2) during the main phases of cell division. In interphase (**a1, a2)**, in prophase (**b1, b2)**, in prometaphase (**c1, c2)**, in metaphase (**d1, d2)**, in anaphase A and B (**e1, e2**, **f1, f2)**, in telophase (**g1, g2)**.

The cell cycle is defined as the series of events that takes place leading up to and including cell division. The cell cycle has two important stages: interphase and mitosis. During interphase, the cell grows in size, doubling its DNA. Mitosis is important for the maintenance of the chromosomal set, in which chromosomes are separated into two identical sets of chromosomes in two daughter cells to maintain the genome’s integrity. Mitosis involves several basic stages: prophase, prometaphase, metaphase, anaphase, and telophase. Fluorescent imaging made major contributions to our understanding of the main phases of mitosis during cell division. Chromosome staining is an essential experimental approach for the studies of chromosomal behavior in dividing cells. We applied the click reaction method to visualize mitotic progression in place of the traditional dye-based imaging. We clearly observed the basic steps of the cell cycle by staining chromosomal DNA with a click reaction (Fig. 6). After a period of cell growth in interphase, the cell enters mitosis and chromosomes begin to condense in prophase. In prometaphase, the next step of mitosis, the nuclear envelope breaks down, and chromosomes congression begins. In metaphase, the chromosomes line up in the cell in their most condensed and coiled stage. During anaphase, we observed that the chromosome pairs divided and moved to opposite poles of the cell. In telophase, the chromosomes continued to separate and were cordoned off into the new nuclei. The key stages of cell division can be visualized by click reactions to stain the chromosomes, suggesting that the technique is useful in studies of cell division.

## Conclusions

First, the click reaction connects the chromosomal DNA and commercial dye molecules and allows for the multicolor staining of chromosomes. The observation of chromosomes in a desired color is possible by changing the fluorescent dye molecules that react with the DNA. Next, we developed a turn-on fluorescent strategy based on the click reaction. Two pro-fluorophore moieties served as light-up reporters to stain chromosomal DNA, which can be used to directly visualize the clear chromosomes in multicolor. Multi-labeling also offers a way to view images in an overlay mode by combination of two or three fluorescence images and allows us to visualize clearly the co-localization images in a multi-pattern. In addition to eliminate the background fluorescence using the turn-on fluorescent strategy by the click reaction, a covalent bond formation between the chromosomal DNA and fluorescent molecules by the click reaction allows that chromosomal DNA imaging is less affected by affinity, environment, or chromosomal structure, which remained a matter of concern from traditional dyes.

Furthermore, we demonstrated that the chromosomes stained by this approach are effective with the FISH technique for detection of telomere DNA at the ends of chromosomes. We further applied this approach to observe several important stages of cell division. We found that the click reaction can be used to directly visualize the key stages of cell division. These results suggest that the click chemistry approach provides a powerful technique for imaging chromosomes for chromosome analysis and genetic diagnostics.

## Methods

### Chemistry

^1^H and ^13^C NMR spectra were measured at 500 MHz on a Bruker AMX spectrometer or 300 MHz on a Bruker (300-AVM) magnetic resonance spectrometer. High-resolution electrospray ionization (ESI) mass spectra were recorded using Exactive Orbitrap mass spectrometer (Thermo Scientific). Data was acquired using Xcalibur software (Thermo Scientific). All samples were dissolved in methanol (LC-MS grade, Wako), and the sample solutions were infused into the ESI source at a flow rate of 20 μL/min by using instrument’s syringe pump. In photography experiments, UV irradiation of 365 nm was achieved with a UV Spot Light Source (Hamamatsu Photonics, 200 W) and UV-D36C filter (Asahi Technoglass). Reaction condition: [EdU] = 2.5 mM, [**1** or **2**] = 2.5 mM, [CuSO_4_] = 25 mM, [Ascorbic acid] = 125 mM, r.t. 2 h. Fluorescent spectra were measured using a JASCO model FP-8200 spectrofluorometer. JASCO Spectra Manager Software was used for data capture and processing of all spectra. The spectra were recorded using a 1-cm path-length cell. For each sample, at least two spectrum scans were accumulated over a wavelength range from 300–650 nm. DMSO-*d*_*6*_ and CDCl_3_ were used as the solvent. Chemical shifts are reported in parts per million shift (δ value) from Me_4_Si (δ 0 ppm for ^1^H) as an internal standard. Coupling constants (*J*) values are given in Hz and are correct to within 0.5 Hz. Signal patterns are indicated as br, broad; s, singlet; d, doublet; t, triplet; q, quartet; sex, sextet; m, multiplet. All reagents were purchased from Sigma-Aldrich, TCI (Tokyo Chemical Industry) or Wako (Wako Pure Chemical Industries). For organic synthesis, reagents of synthesis grade (>98% purity tested by GC) from Sigma-Aldrich, guaranteed reagent (GR) grade from TCI, and special grade from Wako were used, respectively. Thin layer chromatography was performed using TLC Silica gel 60 F_254_ (Merck).

### Synthesis of the pro-fluorophores 1

A mixture of 2, 4, 5-trihydroxy benzaldehyde (3.08 g, 20 mmol), *N*-acetylglycine (2.34 g, 20 mmol), anhydrous sodium acetate (4.9 g, 60 mmol) in acetic anhydride (100 mL) was refluxed under stirring for 4 h. The reaction mixture was poured onto ice to give a yellow precipitate. After filtration, the yellow solid was washed by ice water before it was refluxed in a solution of concentration HCl and ethanol (2:1, 30 mL) for 1 hour, then ice water (40 mL) was added to dilute the solution. The solution was then cooled in an ice bath and sodium nitrite (40 mmol) was added. The mixture was stirred for 5–10 min and sodium azide (60 mmol) was added in portions. After stirring for another 15 min, the resulting precipitate was filtered off, washed with water, and dried under reduced pressure to afford a brown solid pro-fluorophore 1; 2.1 g (48% overall yield).

### Synthesis of the pro-fluorophores 2

First, 3-amino-7-dibuthylaminocoumarin was synthesized from 3-nitro-7-dibuthylaminocoumarin (See Supporting Information). Next, 3-amino-7-dibuthylaminocoumarin (100 mg, 0.43 mmol) was dissolved slowly in HCl aq. (17.2%, 4 mL) at room temperature. Upon cooling to 0–5 °C and addition of a solution of sodium nitrite (30 mg, 0.43 mmol), the reaction mixture was stirred for 1 hour at 0–5 °C. This was followed by the addition of potassium acetate (2 g) in water (5 mL) to adjust the pH of the resulting solution to 4. Sodium azide (57 mg, 0.88 mmol) was added in portions at 0–5 °C, the mixture stirred at 0–5 °C for another 5 h. The precipitated product was rapidly filtered, washed with ice-cold water (10 mL) and dried under vacuum to yield the pro-fluorophore **2** (84 mg, 77%) as a yellow solid.

### Chromosome imaging

For biological assay, all reagents of molecular biology grade from all suppliers were used. HeLa cells were grown at 37 °C and 5% CO_2_ in Dulbecco’s Modified Eagle’s medium (DMEM) containing 10% fetal bovine serum (FBS) and antibiotics (penicillin and streptomycin). 10 μM EdU was added to medium and cells were incubated for 24 h. Colcemid was added to medium and EdU labeled cells were incubated at 37 °C and 5% CO_2_ for 2 h. Cells were washed with PBS, harvested with trypsinization and spinned down. After spinning down the cells, pellet was resuspended in 5 volume of 3:1 (v/v) MeOH/AcOH and incubated at r.t. for 20 min. Cell suspension was fixed on glass slide and treated with 0.5 mg/mL pepsin at r.t. for 10 min. Cells were washed with PBS and were fixed again with 4% of paraformaldehyde (PFA) at r.t. for 10 min. To stain EdU-labeled chromosome, cells were incubated in 24 μM of fluorescent azides (Alexa488-azide and Alexa594-azide) in click reaction buffer (100 mM pH 8.5 Tris-HCl, 1 mM CuSO_4_, and 50 mM ascorbic acid) and incubated at 37 °C and 5% CO_2_ for 30 min under protection from the light. Cells were washed by PBS for several times. To stain chromosomes in multicolor, the cells were stained a second time with 24 μM another fluorescent azide for 30 min. After wash again, chromosomes were observed with AF-6000 (Leica Microsystems) and BZ-9000 Fluorescent microscope. The commercially available reagents and reaction solvents for click reaction were purchased from Invitrogen. Chromosomes were stained by PI and Hoechst 33342 at r.t. for 30 min in dark after washing twice, 10 times, and 20 times by PBS, respectively.

### Chromosome imaging using 1 and 2

EdU-labeled cells were reacted with 24 μM of each pro-fluorophore **1** or **2** in click reaction buffer (Click-iT EdU Imaging Kits from Invitrogen) at 37 °C for 6 h. For multicolor imaging of chromosomal DNA, cells were stained with **1** for 4 h and then with **2** for 1 h. Finally, cells were reacted with Alexa594-azide for 30 min and washed by PBS. Chromosomes were observed with AF-6000 (Leica Microsystems) and BZ-9000 Fluorescent microscope. The excitation and emission filters are 360/40 nm and 470/40 nm for blue fluorescence (compound 1), 480/40 and 527/30 for green fluorescence (compound 2 and Alexa 488) and 546/12 and 600/40 for red fluorescence (Alexa 594). Images are acquired using Leica AF6000 and BZ-II analyzer softwares.

### FISH assay

**1**, **2** and Alexa594-azide stained cells were washed three times with PBS, dehydrated for 5 min in 70% EtOH, 5 min in 95% EtOH and 5 min in 100% EtOH and dried in air. Hybridization buffer (10 mM pH 7.2 Tris-HCl, 0.5% blocking reagent, and 70% formamide) containing 0.1 μM fluorescent labeled PNA (Cy3–(CCCTAA)_3_ for Cy3 labeling and FAM–(CCCTTA)_3_ for FAM labeling) was added to the cells. Then cells were denatured at 80 °C for 3 min and incubated at r. t. for 2 h. After incubation, cells were washed twice for 15 min with washing buffer (10 mM pH 7.2 Tris-HCl, 0.1% BSA, and 70% formamide) and three times for 5 min with washing buffer (0.1 M pH 7.2 Tris-HCl, 0.15 M NaCl, and 0.08% Tween 20). Cells were dehydrated with EtOH in the same procedure above and dried in the air. All procedures were carried out under protection from the light. Labeled cells were observed using AF-6000 (Leica Microsystems) and BZ-9000 Fluorescent microscope.

### Study of cell division

Cells were grown on dish in DMEM supplemented with 10% fetal bovine serum, 1% penicillin, and 1% streptomycin, plated at 30–40% confluence. Remove the culture media, cells were washed twice with 1 × PBS, then added culture media. 10 μM EdU was added to culture media for 24 h. Cell suspensions were fixed on glass dish by 1 mL of 3.7% formaldehyde in PBS for 15 min, washed once with 1 mL of 3% BSA in PBS and treated with 1 mL of 0.005% pepsin at r.t. for 10 min. Cells were washed once and fixed again with 1 mL of 3.7% fixative at r.t. for 10 min. To observe the basic steps of mitosis, chromosomal DNA was stained by click reaction and observed using AF-6000 (Leica Microsystems) and BZ-9000 Fluorescent microscope.

## Additional Information

**How to cite this article**: Ishizuka, T. *et al.* Fluorescence imaging of chromosomal DNA using click chemistry. *Sci. Rep.* **6**, 33217; doi: 10.1038/srep33217 (2016).

## Supplementary Material

Supplementary Information
