# Supplementary material for: Fluorescence imaging of chromosomal DNA using click chemistry
Source: Sci Rep. 2016 Sep 13;6:33217. doi: 10.1038/srep33217 (PMC5020420; doi:10.1038/srep33217)

Supporting Information

Fluorescence imaging of chromosomal DNA using click chemistry

Takumi Ishizuka[1], Hong Shan Liu[1], Kenichiro Ito[2], and Yan Xu[1]*

[1]Division of Chemistry, Department of Medical Sciences, Faculty of Medicine, University of Miyazaki, Japan.

[2]Research Center for Advanced Science and Technology, The University of Tokyo, Japan

*e-mail: xuyan@med.miyazaki-u.ac.jp

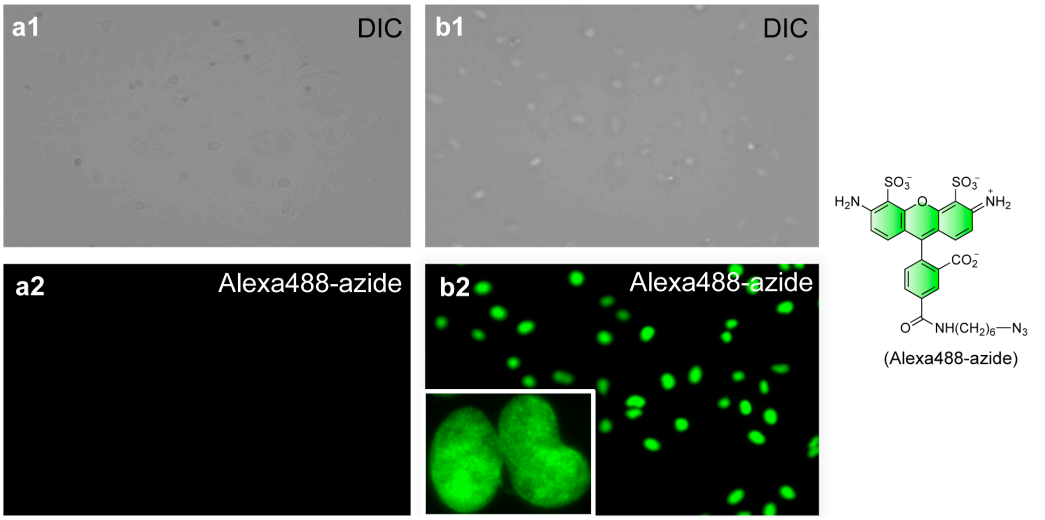

Supplementary Figure 1. Detection of EdU incorporated into the DNA of HeLa cells by fluorescence microscopy. (**a1**, **2**) HeLa cells were incubated in media without EdU. (**b1**, **2**) HeLa cells labeled by incubation overnight with EdU and reacted successively with Alexa488-azide (Chemical structure of Alexa488-azide shows in left). The inset panel is at higher magnification. Cells were imaged by fluorescence microscopy. DIC: Differential interference contrast.

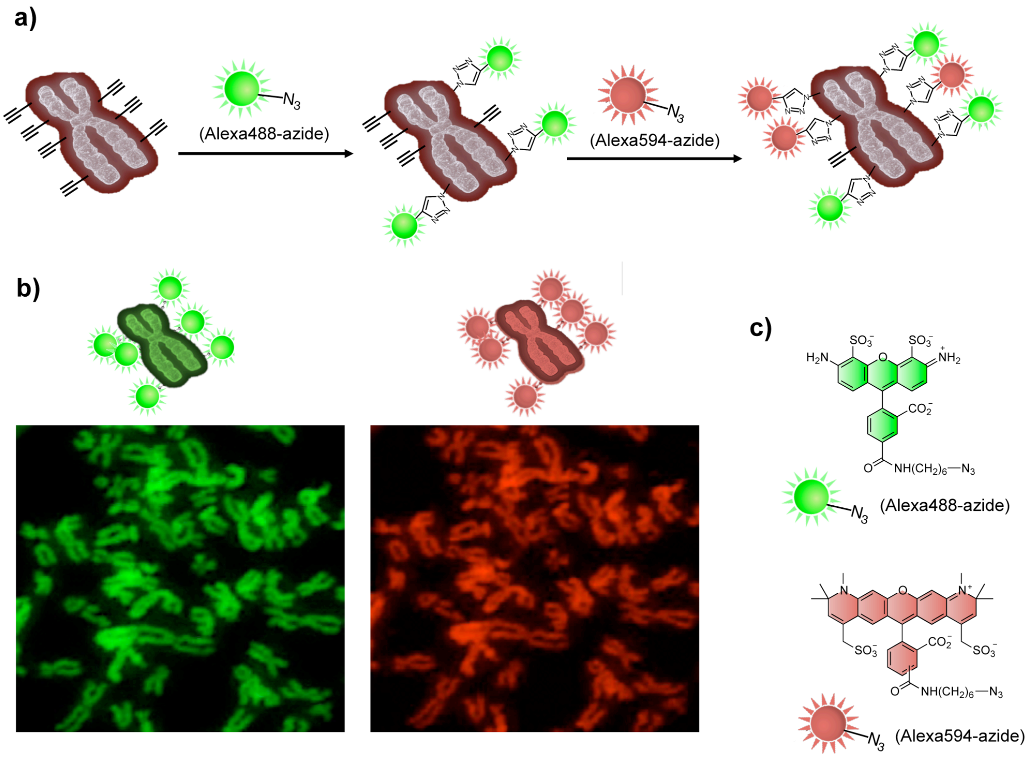

Supplementary Figure 2. Chromosome multicolor staining by click raction. (**a**) Schematic of the click reaction for multicolor staining chromosomal DNA. First, EdU-labeled chromosomes were reacted with Alexa488-azide. Subsequently, the chromosomes were stained with Alexa594-azide. (**b**) Chromosomes were stained with Alexa488-azide (green) and Alexa-594 azide (red), respectively. Observed by fluorescence microscopy. (**c**) Chemical structures of Alexa488-azide and Alexa594-azide.

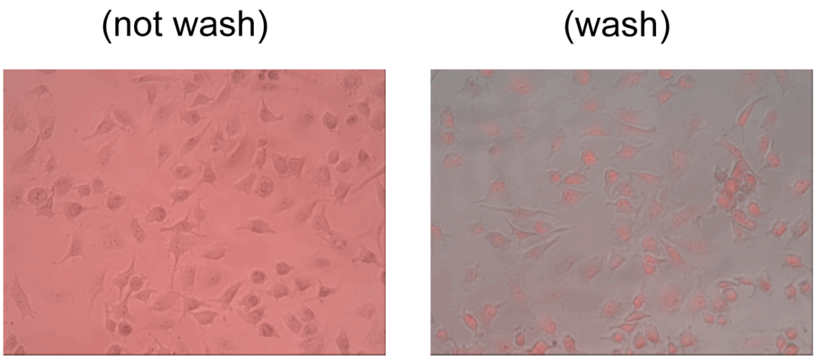

Supplementary Figure 3. A comparison between cells with and without washing step. EdU-labeled cells were reacted with fluorescent azide (Alexa594-azide). Washing step with PBS buffer was used to remove unbound fluorescent dyes (right), eliminating the wash step (left) as a control. Fluorescence was detected with microscope.

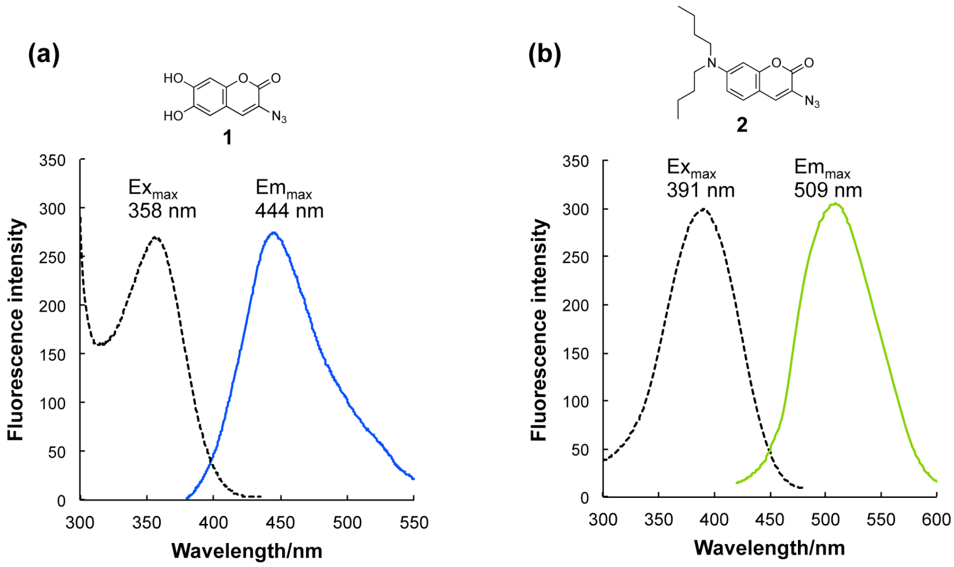

Supplementary Figure 4. Excitation and emission spectra of compounds **1** and **2**. The excitation and emission maximum values (Ex_{max} and Em_{max}) for the two compounds are indicated.

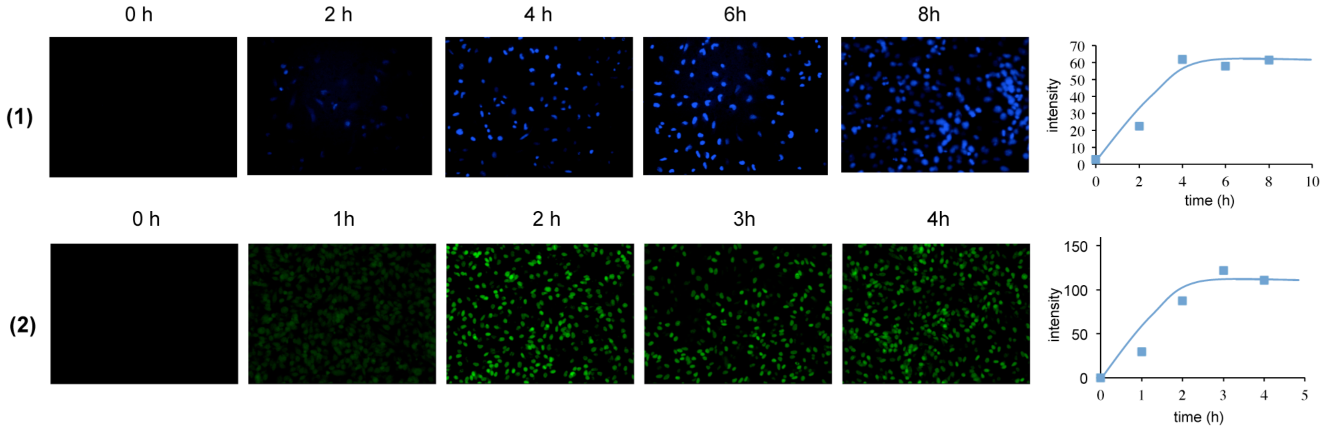

Supplementary Figure 5. The time course of DNA light-up labeling in cells using **1** or **2**. Quantitation of fluorescent intensity plotted as a percentage (three experiments).

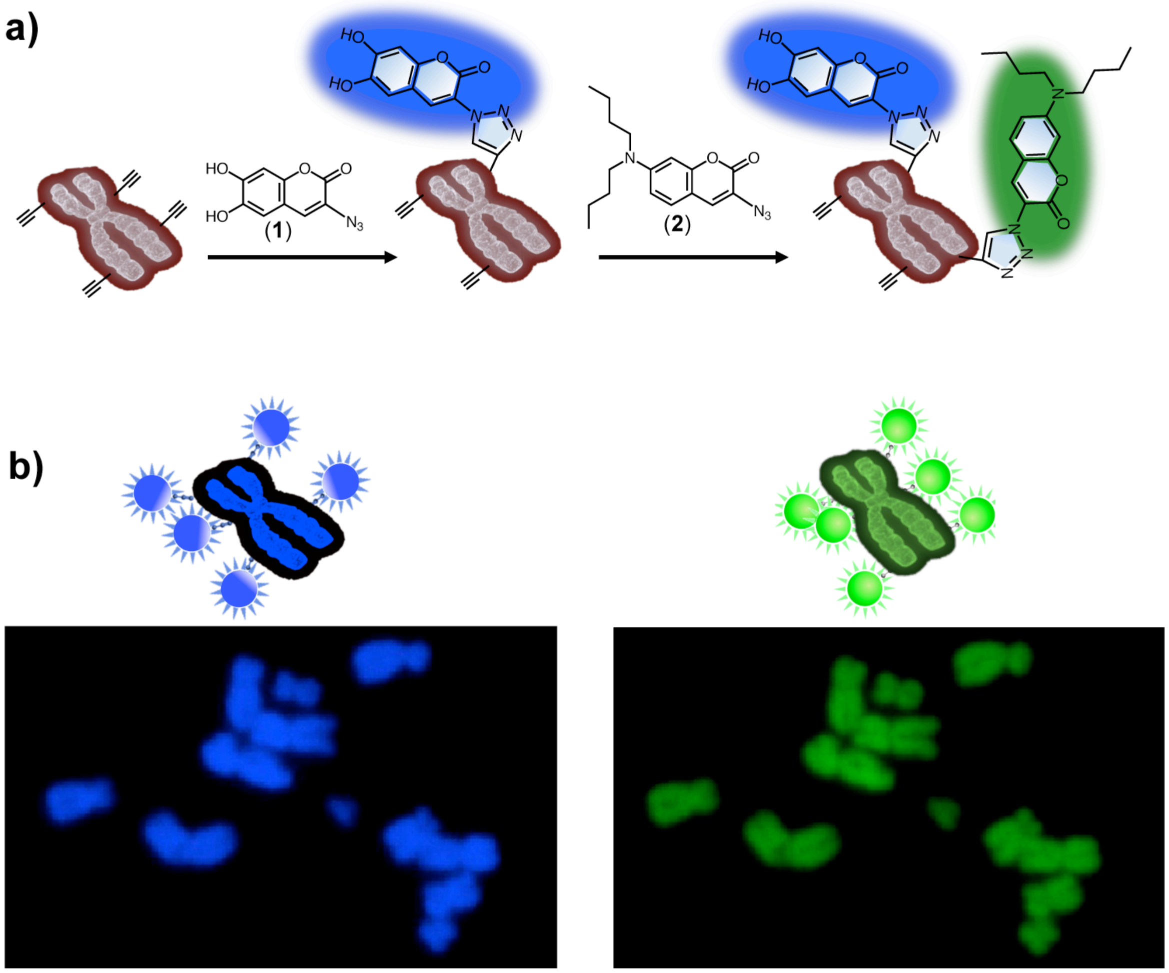

Supplementary Figure 6. Chromosome multicolor staining by using **1** and **2**. (**a**) Schematic of the click reaction for multicolor staining chromosomal DNA. First, EdU-labeled chromosomes were reacted with pro-fluorophores **1**. Subsequently, the chromosomes were stained with pro-fluorophores **2**. (**b**) Chromosomes were stained with pro-fluorophores **1** (blue) and **2** (green), respectively. Observed by fluorescence micr

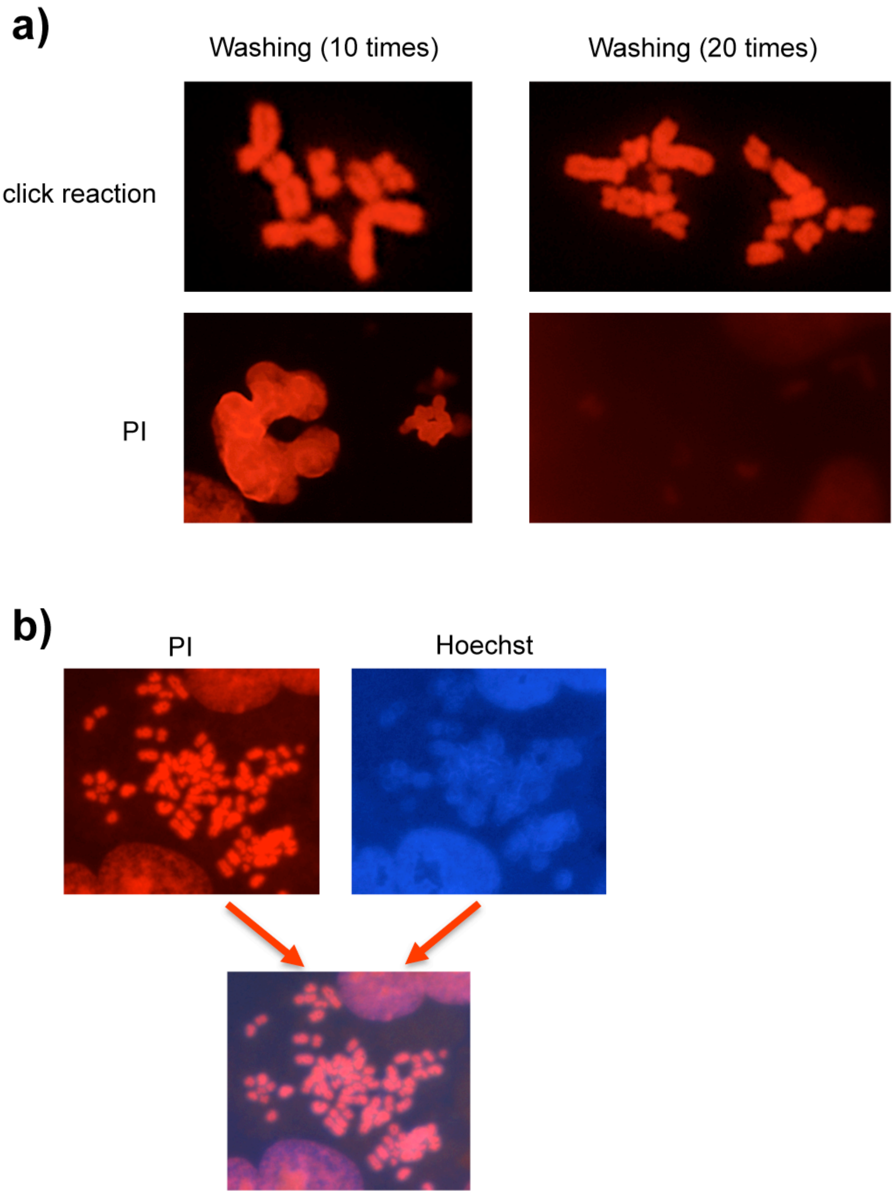

Supplementary Figure 7. Comparison experiments of traditional dye and click reaction straining. (**a**) First, chromosomes were stained by click reaction (Alexa-594 azide in red) and PI, respectively. Next, the chromosomes were washed with PBS buffer in 10 and 20 times. The chromosomal DNA strained by click reaction method does not occur any changes with the repetitive wash steps (10 and 20 times). However, the chromosomal DNA strained PI binding shows unclear chromosomal DNA signals with repetitive wash steps in10 times, the wash steps with 20 times result in a failed observation in DNA staining. (**b**) Chromosome multicolor staining by using traditional dyes, PI and Hoechst. (**a**) First, chromosome DNA was bound with PI. Subsequently, the chromosome DNA was bound with Hoechst. The combination of PI and Hoechst images is represented.

Synthesis of the pro-fluorophores 1 and 2. The pro-fluorophores **1** and **2** were prepared according to the following scheme.

Scheme 1.

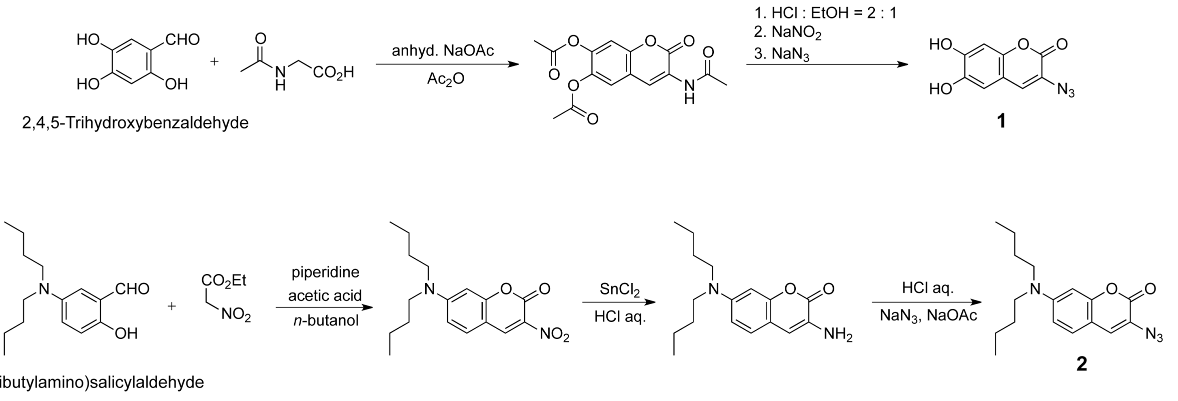

Pro-fluorophores 1. ^{1}H NMR and ^{13}C NMR spectra were recorded on a Bruker (300-AVM) magnetic resonance spectrometer. ^{1}H NMR (DMSO-d_6, 300 MHz) δ 6.77 (s, 1H), 6.93 (s, 1H), 7.54 (s, 1H), 9.46 (s, 1H), 10.16 (s, 1H). ^{13}C NMR (DMSO-d_6, 75 MHz) δ 102.51, 110.92, 111.75, 121.13, 128.01, 143.43, 145.85, 149.43, 157.50. HRMS (ESI) for $C_9H_4O_4N_3$ $[M-H]^-$: Calcd. 218.0196; Found. 218.0199.

3-Nitro-7-dibuthylaminocoumarin

A mixture containing *n*-butanol (20 mL), 4-(dibutylamino)salicylaldehyde (1.4 g, 7.2 mmol), ethyl nitroacetate (0.8 mL, 7.2 mmol), molecular sieves 4 Å (100 mg), piperidine (0.1 mL) and acetic acid (0.2 mL) was refluxed for a period of 24 h. Upon cooling to room temperature, a bright yellow solid formed, which was collected and dissolved in DMF (15 mL) at 80 °C. It was filtered again to remove the molecular sieves. The filtrate, upon addition to 100 ml of ice-cold water, yielded compound 3-Nitro-7-dibuthylaminocoumarin (1.40 g, 78 %) as a bright yellow solid. ^{1}H NMR (DMSO-d_6, 500 MHz) δ 0.92 (t, J = 7.0 Hz, 6H), 1.35 (m, 4H), 1.54 (m, 4H), 3.47 (m, 4H), 6.61 (s, 1H), 6.90 (d, J = 8.6 Hz, 1H), 7.73 (d, J = 9.0 Hz, 1H), 9.03 (s, 1H). ^{13}C NMR ($CDCl_3$, 125 MHz) δ 13.85, 20.19, 29.27, 51.54, 97.00, 106.21, 111.28, 132.45, 143.36, 153.57, 154.92, 158.71, 163.48. ESI-MS (*m/z*) $[M+H^+]^+$ calcd for $C_{13}H_{14}N_2O_4$, 319.16; found 319.12.

3-Amino-7-dibuthylaminocoumarin

In a 25 mL round bottomed flask equipped with a magnetic stirrer, were placed in order, 37.4 % HCl (5 mL), stannous chloride dihydrate (1.6 g, 7.12 mmol). To this suspension compound 3-Nitro-7-dibuthylaminocoumarin (0.25 g, 0.95 mmol) was added at room temperature in small portions, over a period of thirty minutes. Stirring was continued for 4 h before the solution was poured onto 20 g of ice and made alkaline using sodium hydroxide solution (5 M) at 15 °C using an ice-water bath. The resulting suspension was then extracted with diethyl ether (2 × 25 mL). The organic layer was washed with water (50 mL), dried over anhydrous Na_2SO_4 and concentrated to a pasty residue which upon triturating using hexane yielded compound 3-Amino-7-dibuthylaminocoumarin (0.15 g, 66 %) as a pale yellow solid. ^{1}H NMR ($CDCl_3$, 500 MHz) δ 0.95 (t, J = 7.6 Hz, 6H), 1.33 (sex, J = 7.6 Hz, 4H), 1.56 (quin, J = 7.6 Hz, 4H), 3.26 (t, J = 7.6 Hz, 4H), 3.84 (br s, 2H), 6.48 (d, J = 2.4 Hz, 1H), 6.52 (dd, J = 2.4, 8.7 Hz, 1H), 6.69 (s, 1H), 7.08 (d, J = 8.7 Hz, 1H). ^{13}C NMR ($CDCl_3$, 125 MHz) δ 13.98, 20.30, 29.32, 50.99, 98.02, 109.35, 109.54, 114.57, 125.88, 127.44, 147.93, 151.61, 160.40. ESI-MS (*m/z*) $[M+H^+]^+$ calcd for $C_{17}H_{24}N_2O_2$, 289.18; found 289.19.

Pro-fluorophores 2. ^{1}H NMR ($CDCl_3$, 500 MHz) δ 0.97 (t, *J* = 7.6 Hz, 6H), 1.37 (sex, *J* = 7.6 Hz, 4H), 1.58 (quin, *J* = 7.6 Hz, 4H), 3.31 (t, *J* = 7.6 Hz, 4H), 6.48 (d, *J* = 2.2 Hz, 1H), 6.56 (dd, *J* = 2.2, 8.8 Hz, 1H), 7.10 (s, 1H), 7.17 (d, *J* = 8.8 Hz, 1H). ^{13}C NMR ($CDCl_3$, 125 MHz) δ 13.94, 20.26, 29.24, 51.03, 97.48, 107.94, 109.54, 119.49, 127.65, 128.04, 150.24, 154.13, 158.46. HRMS (ESI) for $C_{17}H_{23}O_2N_4$ $[M+H]^+$: Calcd. 315.1816; Found. 315.1813.

HRMS spectrum of compound **1** (negative mode)

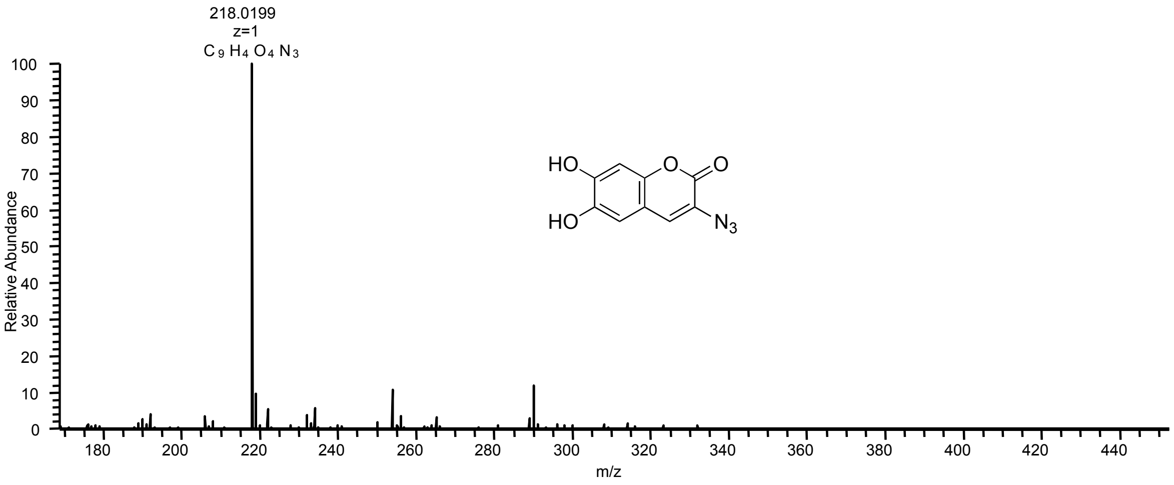

HRMS spectrum of compound **2** (positive mode)

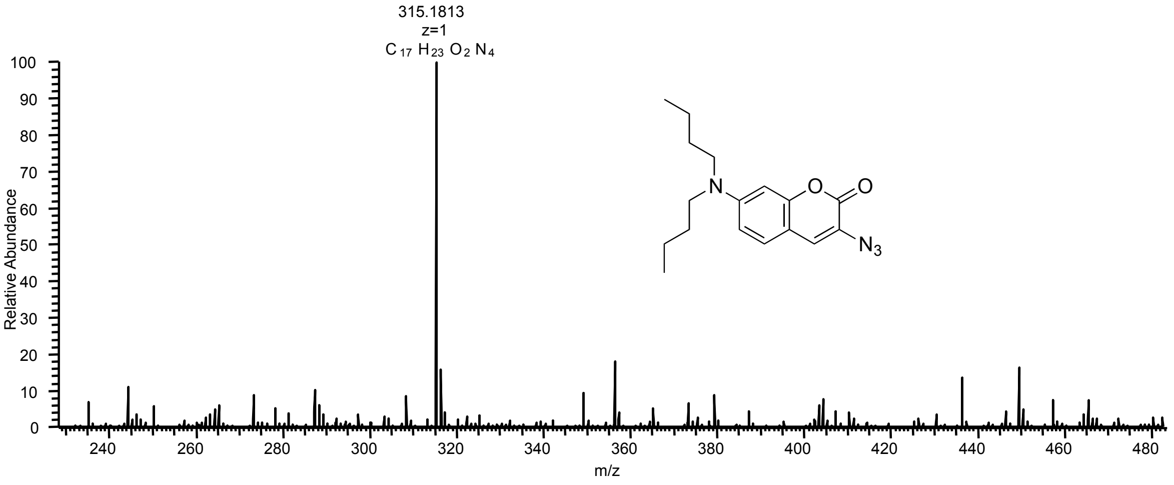

^{1}H-NMR spectrum of compound **1**

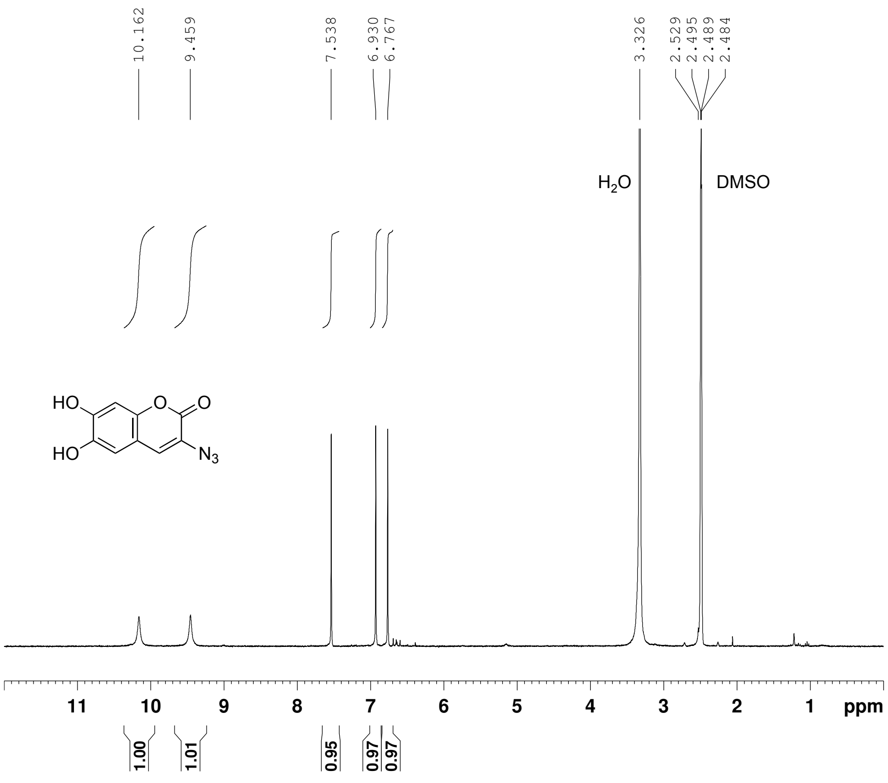

^{13}C-NMR spectrum of compound **1**

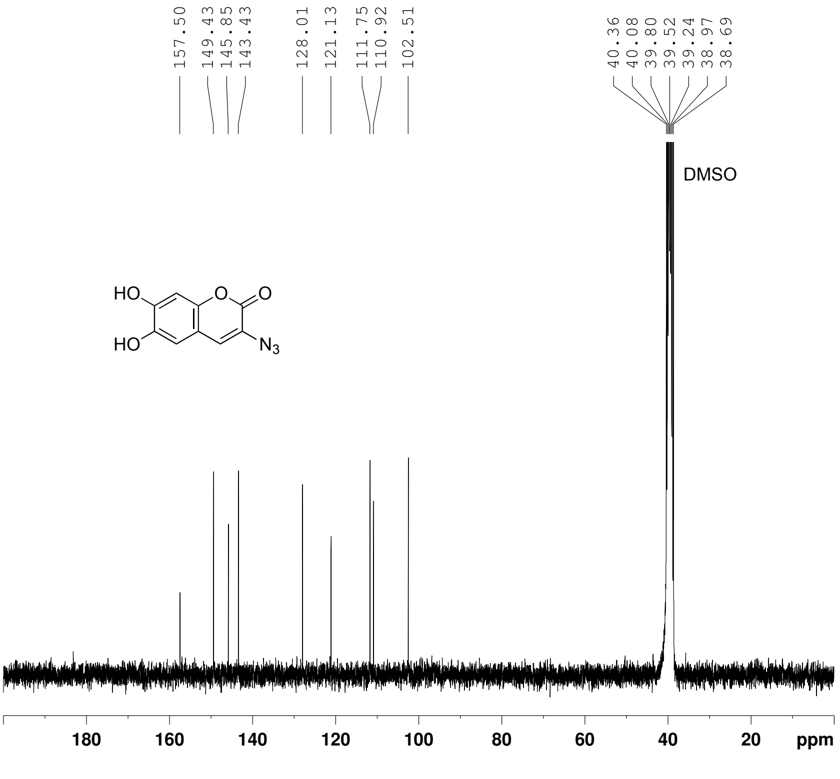

^{1}H-NMR spectrum of compound 3-Nitro-7-dibuthylaminocoumarin

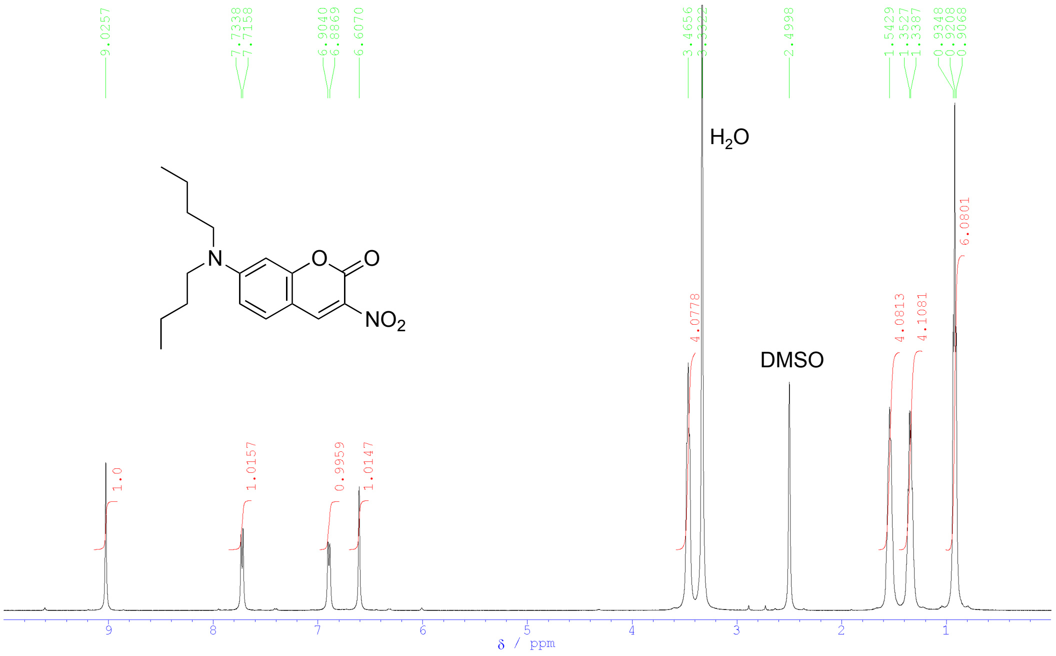

^{13}C-NMR spectrum of compound 3-Nitro-7-dibuthylaminocoumarin

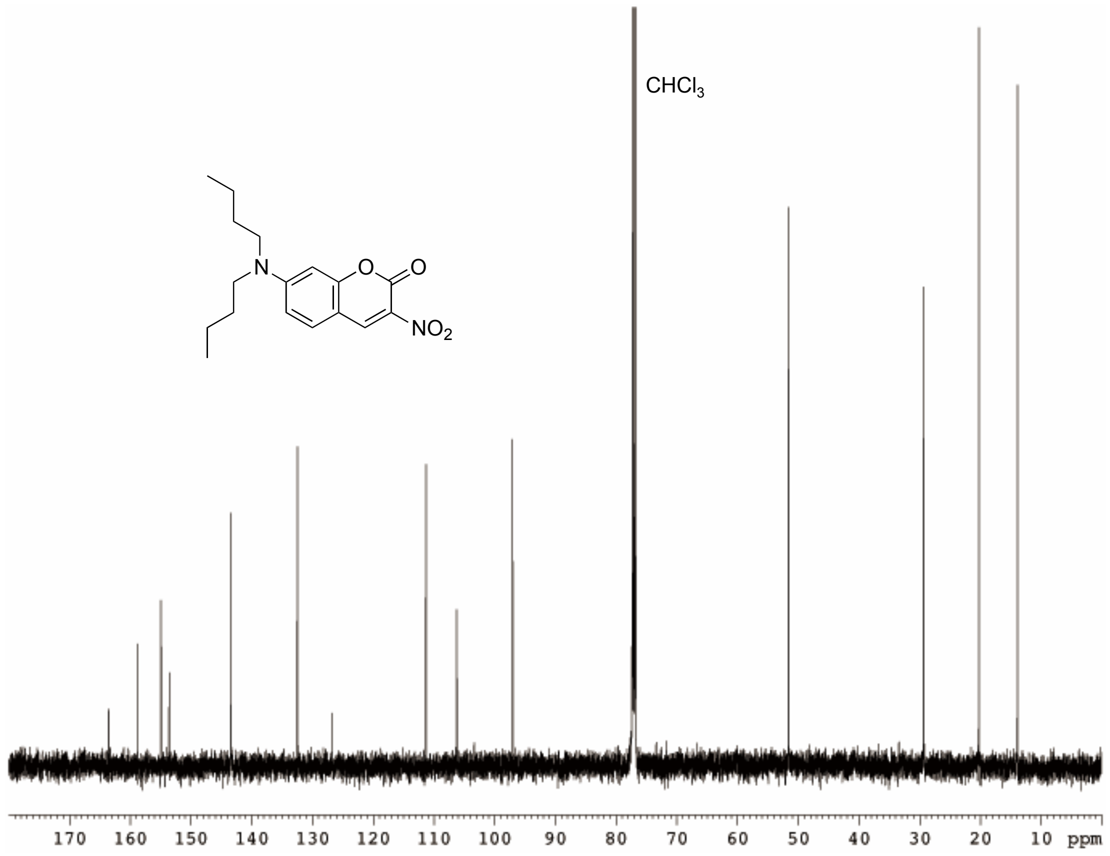

^{1}H-NMR spectrum of compound 3-Amino-7-dibuthylaminocoumarin

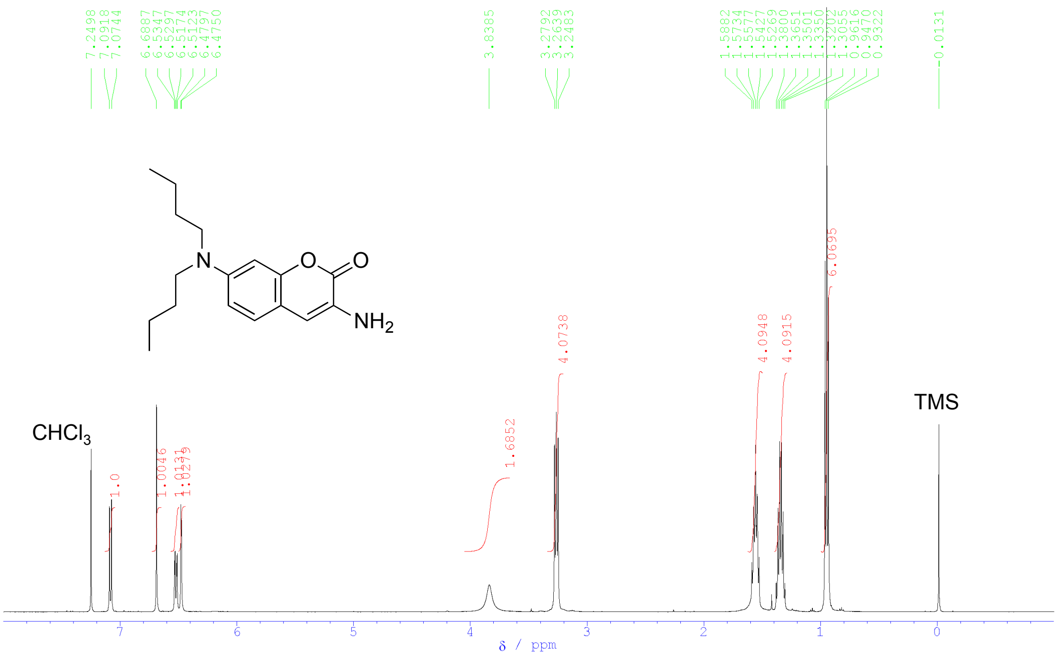

^{13}C-NMR spectrum of compound 3-Amino-7-dibuthylaminocoumarin

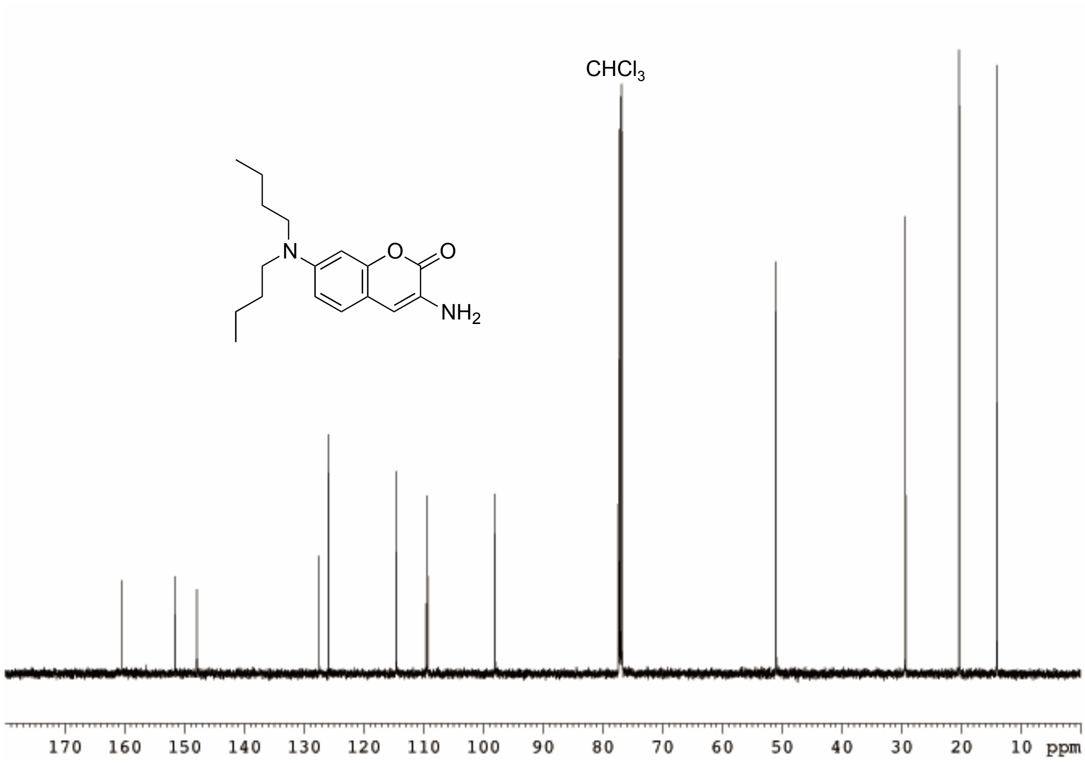

^{1}H-NMR spectrum of compound **2**

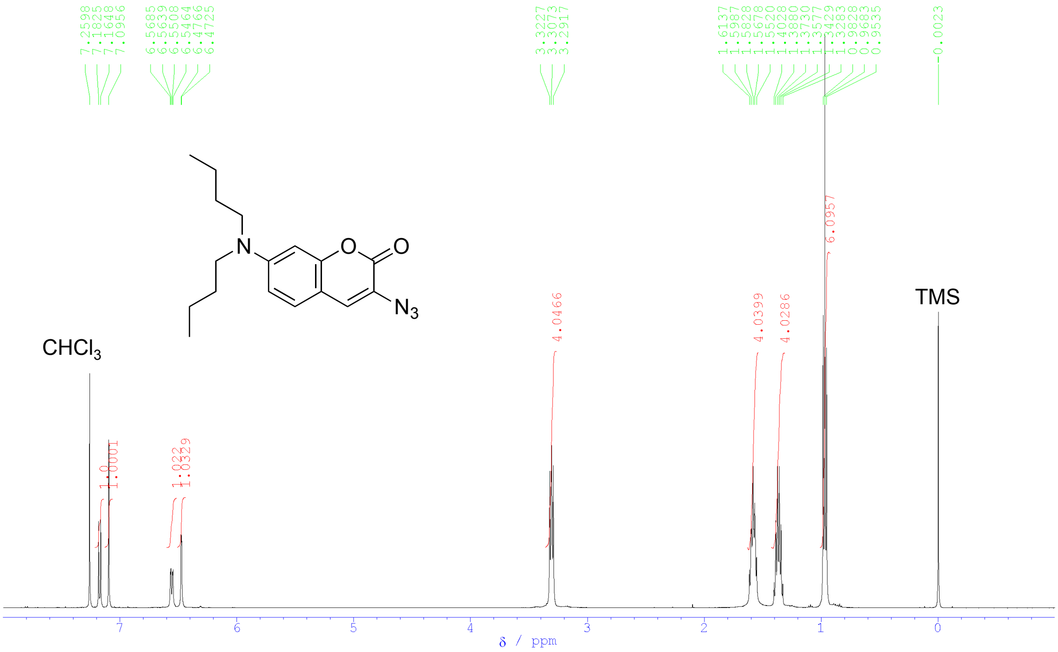

^{13}C-NMR spectrum of compound **2**

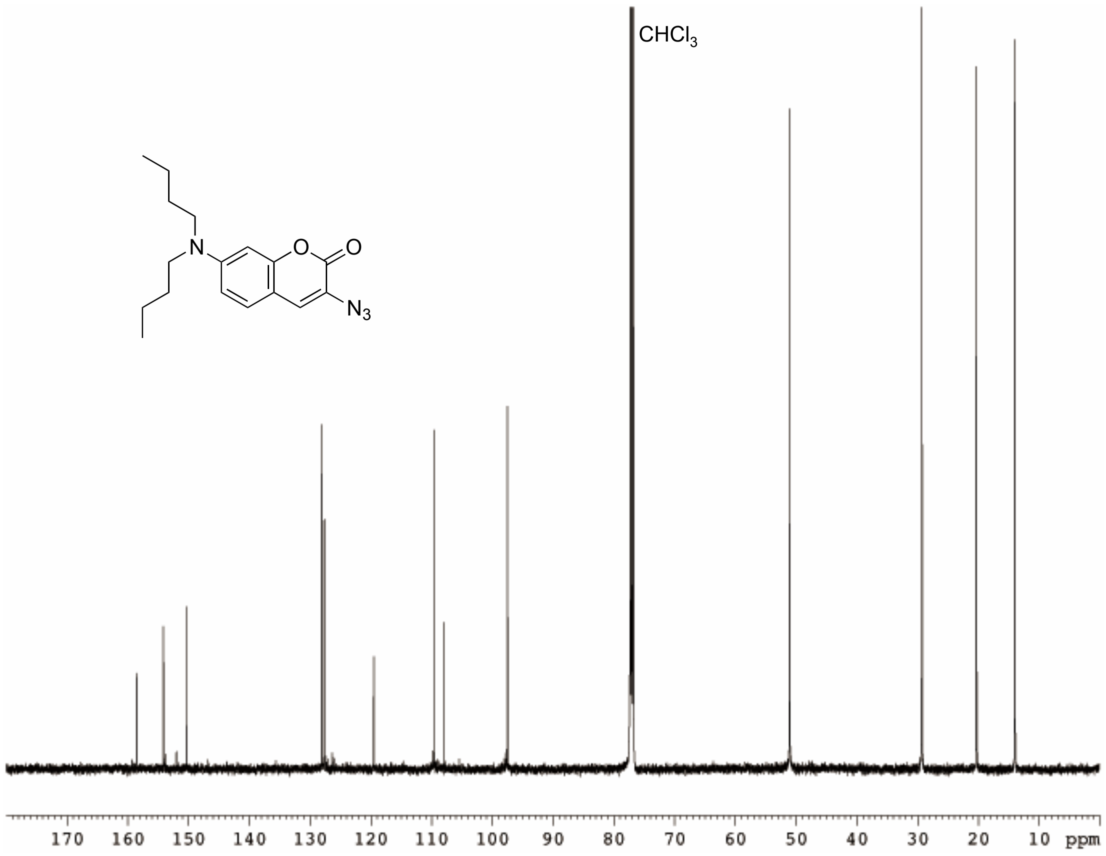

Supplement: Supplementary Information [file srep33217-s1.pdf]
